# Inhibition of c-MYC with involvement of ERK/JNK/MAPK and AKT pathways as a novel mechanism for shikonin and its derivatives in killing leukemia cells

**DOI:** 10.18632/oncotarget.5380

**Published:** 2015-10-09

**Authors:** Qiaoli Zhao, Andreana N. Assimopoulou, Sabine M. Klauck, Harilaos Damianakos, Ioanna Chinou, Nadine Kretschmer, José-Luis Rios, Vassilios P. Papageorgiou, Rudolf Bauer, Thomas Efferth

**Affiliations:** ^1^ Department of Pharmaceutical Biology, Institute of Pharmacy and Biochemistry, Johannes Gutenberg University, Mainz, Germany; ^2^ Department of Chemical Engineering, Aristotle University of Thessaloniki, Thessaloniki, Greece; ^3^ Working Group Cancer Genome Research, German Cancer Research Center (DKFZ) and German Cancer Consortium (DKTK), National Center for Tumor Diseases (NCT), Heidelberg, Germany; ^4^ Faculty of Pharmacy, University of Athens, Athens, Greece; ^5^ Department of Pharmacognosy, Institute of Pharmaceutical Sciences, University of Graz, Graz, Austria; ^6^ Department de Farmacologia, Facultat de Farmàcia, Universitat de València, Valencia, Spain

**Keywords:** shikonin and its derivatives, c-MYC, ERK/JNK/MAP kinases, AKT pathway, acute leukemia

## Abstract

Leukemia remains life-threatening despite remarkable advances in chemotherapy. The poor prognosis and drug resistance are challenging treatment. Novel drugs are urgently needed. Shikonin, a natural naphthoquinone, has been previously shown by us to be particularly effective towards various leukemia cell lines compared to solid tumors. However, the underlying mechanisms are still poorly understood. Here, we investigated shikonin and 14 derivatives on U937 leukemia cells. Four derivatives (isobutyrylshikonin, 2-methylbutyrylshikonin, isovalerylshikonin and β,β-dimethylacrylshikonin) were more active than shikonin. AnnexinV-PI analysis revealed that shikonins induced apoptosis. Cell cycle G1/S check point regulation and the transcription factor c-MYC, which plays a vital role in cell cycle regulation and proliferation, were identified as the most commonly down-regulated mechanisms upon treatment with shikonins in mRNA microarray hybridizations. Western blotting and DNA-binding assays confirmed the inhibition of c-MYC expression and transcriptional activity by shikonins. Reduction of c-MYC expression was closely associated with deregulated ERK, JNK MAPK and AKT activity, indicating their involvement in shikonin-triggered c-MYC inactivation. Molecular docking studies revealed that shikonin and its derivatives bind to the same DNA-binding domain of c-MYC as the known c-MYC inhibitors 10058-F4 and 10074-G5. This finding indicates that shikonins bind to c-MYC. The effect of shikonin on U937 cells was confirmed in other leukemia cell lines (Jurkat, Molt4, CCRF-CEM, and multidrug-resistant CEM/ADR5000), where shikonin also inhibited c-MYC expression and influenced phosphorylation of AKT, ERK1/2, and SAPK/JNK. In summary, inhibition of c-MYC and related pathways represents a novel mechanism of shikonin and its derivatives to explain their anti-leukemic activity.

## INTRODUCTION

Acute leukemia is a fast-growing clonal malignancy of the hematopoietic system that typically leads to death within weeks or months, if left untreated. Based on the type of white blood cell, it is classified into acute myeloid leukemia (AML) and acute lymphoblastic leukemia (ALL), which together account for approximately one-third of cancers diagnosed in children [1]. Despite dramatic improvement in ALL and AML treatment, acute leukemia remains the most common cause of cancer-related death in children due to the poor prognosis for certain high-aggressive groups of leukemia and drug resistance in relapsed or refractory patients [2, 3]. Therefore, deeper insight into the molecular mechanisms of leukemia progression is required for the development of novel anti-leukemia agents [4].

The proto-oncogene *c-MYC* encodes a basic helix-loop-helix leucine zipper (bHLH-Lz) transcription factor, which plays a pivotal role in cell proliferation, metabolism, differentiation, apoptosis and tumorigenesis by transcription and activation of downstream target genes [5]. For example, cell cycle progression from the G0/G1 into the S phase is tightly controlled by c-MYC by regulating the expression of cyclins, cyclin dependent kinases (CDK), CDK inhibitors and the pRb-binding transcription factor E2F [6]. About 50% of both blood-borne and solid tumors over-express c-MYC protein, which is usually correlated with poor prognosis due to promoting tumor growth and resistance to drugs [7]. c-MYC deregulation is closely associated to hematopoietic neoplasia [8, 9]. In fact, the retroviral form, *v-myc* was first discovered to cause myelocytomatosis in chicken and the oncogene was named after this tumor [7]. Later, the cellular pendant, *c-MYC*, was found to be translocated in aggressive Burkitt's lymphoma. The important role for *c-Myc* on leukemogenesis was subsequently confirmed in animal models. Conditional overexpression *c-Myc* in hematopoietic cells in transgenic mice led to the formation of malignant T-cell lymphomas and acute myleoid leukemias, which were reverted by inactivation of the *c-MYC* transgene [10, 11]. Later on, mounting evidence has been accumulated showing that the c-MYC protein is a key player in hematopoiesis and leukemia [9]. Recently, c-MYC is closely correlated to drug resistance in leukemia cells. Leukemic cell lines resistant to cytarabine displayed a c-MYC-dependent overexpression of the natural killer (NK) group 2, member D (NKG2D) ligands (NKG2DL) UL-16 binding proteins 1–3 (ULBP1-3) [12]. Up-regulated expression of c-MYC in leukemia cells promoted the colony formation ability and maintained poor differentiation leading to drug resistance [5]. In addition, c-MYC contributed to microenvironment-mediated drug resistance in AML [13]. All these studies speak for the potential of c-MYC as therapeutic target. Inactivation of c-MYC represents as a novel approach to improve clinical outcome and prognosis in leukemia treatment.

c-MYC heterodimerizes with its activation partner MAX, which is also a member of bHLH-LZ protein family, to recognize the specific E-box CACGTG DNA sequences in the promoters of its target genes. Thereby, it exerts most of its fundamental biological activities. A straightforward strategy to inhibit c-MYC functions is to block its DNA binding activity by either interfering with c-MYC–MAX dimerization or disrupting the interaction of transcriptionally active c-MYC–MAX dimers with DNA [14, 15]. In this context, several small-molecule c-MYC inhibitors have been identified from large chemical libraries. For some of them, *e.g*. 10058-F4 and 10074-G5, the actual binding modes have been elaborately illustrated [16, 17]. Another mechanism of c-MYC inactivation involves the interference of signal transduction pathways that down-regulate c-MYC expression. Many signaling pathways, including phosphatidylinositol 3-kinase (PI3K)/AKT, Ras-Raf-MEK-ERK mitogen-activated protein kinase (MAPK), regulate *c-MYC* mRNA expression and promote c-MYC stability [18, 19]. Marampon *et al* demonstrated that the inhibition of the MEK/ERK pathway dramatically decreased c-MYC expression and thus inhibited in cancer cell growth [20]. Although several small molecules have been described as c-MYC inhibitors, none of them is clinically used as of yet. Therefore, novel c-MYC-targeting drugs are urgently needed.

Natural products are a valuable resource for anticancer agents. Previously, we tested the cytotoxicity of shikonin, a natural naphthoquinone derived from the roots of the Chinese herb *Lithospermum erythrorhizon, Arnebia euchroma* and *Onosma paniculata* [21–23], on a panel of tumor cell lines, including both hematopoietic and solid cancer cell lines [24, 25]. Leukemia cell lines were more sensitive to shikonin compared to solid tumor cell lines, especially the acute myelocytic leukemia cell line U937 [25]. However, the exact mechanisms underlying shikonin-induced leukemia cell death remain unclear. Therefore, we investigated the mode of action on leukemia cells in the present study. The cytotoxic effect and the death mode of shikonin and 14 derivatives in U937 were first examined. Subsequent microarray-based gene expression profiling for shikonin and four most active derivatives indicated that *c-MYC* was commonly deregulated. This result was validated by Western blot analysis and DNA-binding activity assays. *In silico* molecular docking revealed that shikonin and its derivatives bound to c-MYC at the same pharmacophores as the known c-MYC inhibitors 10058-F4 and 10074-G5 with comparable binding energy. Meanwhile AKT, and ERK1/2, JNK/MAPK signaling pathways were also involved in shikonin-induced c-MYC inactivation in U937 cells. Moreover, the exquisite activity of shikonin in U937 cells has been confirmed in other acute leukemia cell lines, implying inhibition of c-MYC as a general mechanism of shikonin and derivatives towards leukemia cells.

## RESULTS

### Cytotoxicity of shikonin and derivatives towards U937 leukemia cells

**Figure 1:**
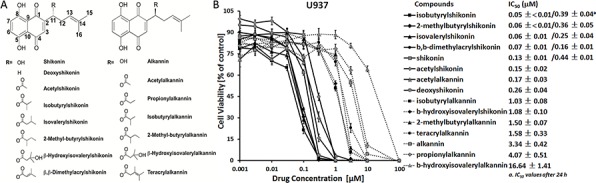
**A.** Chemical structures of shikonin and 14 derivatives. **B.** Cytotoxicity of shikonin and derivatives towards U937 leukemia cells. Cells were treated with varying concentrations of shikonin or 14 derivatives and cell viability was measured by resazurin assay after 24 h or 72 h. Representative dose-response curves and IC_50_ values (mean ± SEM) of 72 h treatment for shikonin and 14 derivatives are shown. The IC_50_ values for shikonin and four derivatives after 24 h are also displayed in parallel. Results are mean values and standard deviation of three independent experiments with each 6 parallel measurements.

Previously we reported the sensitivities of a panel of different cell lines to shikonin and found that the U937 histiocytic leukemia cell line was the most sensitive one [25]. Thereby, this cell line was used for screening the cytotoxicity of shikonin and 14 shikonin derivatives. The dose-response curves and IC_50_ values of 72 h treatment with varying concentrations of shikonin and derivatives are summarized in Figure 1B. Four compounds, *i.e*. isobutyrylshikonin, 2-methylbutyrylshikonin, isovalerylshikonin and β, β-dimethylacrylshikonin, showed stronger effects than shikonin itself. Therefore, these derivatives were further analyzed together with shikonin for their molecular mechanisms against leukemia cells. The IC_50_ values for shikonin and these four derivatives after 24 h were also measured by resazurin assay. The other derivatives were less toxic than shikonin towards U937 cells. Furthermore, it was interesting that shikonin and its homochiral derivatives were more active than their enantiomers, *e.g*., the IC_50_ values of isobutyrylshikonin (0.05 μM), 2-methylbutyrylshikonin (0.06 μM) and β-hydroxyisovalerylshikonin (1.08 μM) were much less than those of their corresponding enantiomers, isobutyrylalkannin (1.03 μM), 2-methylbutyrylalkannin (1.50 μM) and β-hydroxyisovalerylalkannin (16.64 μM).

### Assessment of cell death induced by shikonins as measured by flow cytometry

**Figure 2:**
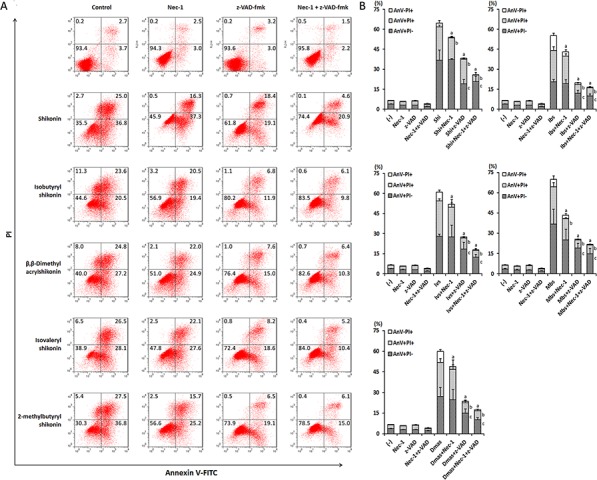
Modes of cell death induced by shikonin and its derivatives in U937 cells **A.** Representative dot plots of flow cytometry analysis after treatment of U937 cells with 50 μM necrostatin-1 (Nec-1) or z-VAD-fmk (z-VAD) 1 h prior to co-incubation with IC_50_ concentrations of shikonin or its derivatives for 24 h. A dual staining with annexin V-FITC/PI was performed. The values indicate the percentage of cells in each region. **B.** The digitalized graphs of the means ± SEM of three independent experiments one is shown in Fig. 2A (*n* = 3). ^a^, *p* < 0.05 *vs.*shikonin or its derivatives AnV-PI+; ^b^, *p* < 0.05 vs. shikonin or its derivatives AnV+PI+; ^c^, *p* < 0.05 vs. shikonin or its derivatives AnV+PI-, calculated by two-tailed Student's *t* test. AnV-PI+, annexin V − /PI+ (late necrosis); AnV+PI+, annexin V+/PI+ (late apoptosis or early necrosis); AnV+PI-, annexin V+/PI− (early apoptosis). Shi, shikonin; Ibs, isobutyrylshikonin; Dmas, β,β-dimethylacrylshikonin; Ivs, isovalerylshikonin; Mbs, 2-methylbutyrylshikonin.

To further investigate death modes caused by shikonin and derivatives in U937 cells, we performed flow cytometry with annexin V and PI double staining in U937 cells treated with and without shikonin and four derivatives in the presence or absence of the specific necroptosis inhibitor Nec-1 and the caspase apoptosis-specific inhibitor z-VAD-fmk. As shown in Figure 2, pretreatment with Nec-1 reduced necrosis (annexin V-/PI+) and partly late apoptosis (annexin V+/PI+), but not early apoptosis (annexin V+/PI-). By contrast, z-VAD-fmk attenuated early and late apoptosis and resulted in more cell viability than Nec-1, indicating that low concentrations of shikonin or its four derivatives mainly induced cell death by caspase-dependent apoptosis. However, the most effective inhibition of cell death by shikonin and its derivatives was achieved by the combination of Nec-1 and z-VAD-fmk, suggesting that necroptosis, as additional mode of death may also contribute to cell death.

### Gene expression profiling of shikonin and derivatives revealed MYC as novel common molecular key player

Gene expression analyses were performed to get deeper insights into the cytotoxic activity of shikonins. U937 cells were treated 24 h with IC_50_ values of shikonin, isobutyrylshikonin, 2-methylbutyrylshikonin, isovalerylshikonin, β,β-dimethylacrylshikonin or DMSO solvent control, respectively. Then, total RNA was isolated for transcriptome-wide microarray analysis.

**Figure 3:**
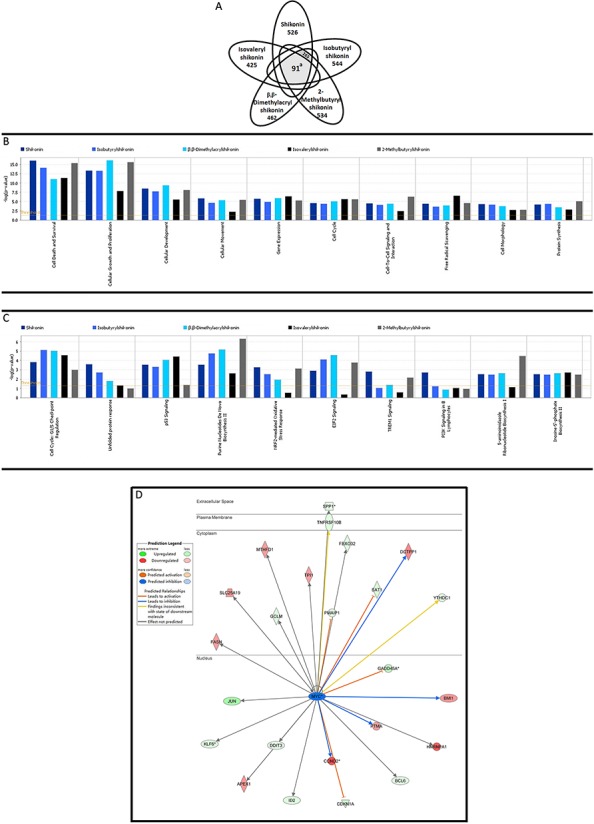
Gene expression profiling of U937 cells upon treatment with shikonin and its derivatives **A.** Venn diagrams: Numbers of genes deregulated after 24 h. Only molecules with fold changes ≥ ± 1.65 are presented in the diagram. ^a^, *p* < 0.0001, indicating the probability of 91 genes occurred by chance, calculated using Monte Carlo simulation method by R language. **B** and **C.** Pathway analyses: Top cellular functions and canonical pathways affected by shikonin and its derivatives examined by mRNA microarray hybridization. *P*-values were calculated using right-tailed Fisher's exact test. **D.** Deregulated genes under the influence of MYC as common upstream regulator inhibited by shikonin and its derivatives.

The numbers of deregulated genes upon treatment with shikonin compounds were visualized as Venn diagram (Figure 3A). Remarkably, about 18% of the genes were present in the datasets of all five compounds. If four of five compounds were taken into account, 265 genes are commonly differentially expressed between treated and untreated cells. Through web-based gene ontology (GO) enrichment analysis, the identified 91 co-regulated genes were assigned to multiple GO terms categorized with the biological process, cellular component and molecular function (Supplement table 1). A ranking list of deregulated genes is shown in Table 1. Remarkably, *MYC* was the most commonly down-regulated gene among all five compounds. Six commonly deregulated genes including *MYC* were quantified by real-time RT-PCR to technically validate the microarray results. The correlation coefficients (R-values) between mRNA expression values determined by microarray hybridization and real-time RT-PCR were in the range of 0.80 to 0.93 for each compound (Pearson correlation test). Importantly, this indicated a high degree of concordance between the data obtained from the two different methods (Table 2).

**Table 1:** Top up- and down regulated molecules in U937 cells upon treatment with shikonin and its derivatives for 24 h

	Shikonin		Isobutyrylshikonin		β,β-Dimethylacrylshikonin		Isovalerylshikonin		2-Methylbutyrylshikonin	
	Gene	Fold change	Gene	Fold change	Gene	Fold change	Gene	Fold change	Gene	Fold change
Top up-regulated genes	RNU11	18,5	LY96	12,4	IER3	10,6	RMRP	74,0	LY96	11,2
	IER3	9,6	IER3	10,5	RNU11	10,6	RNU11	73,3	IER3	9,5
	LY96	9,0	JUN	7,1	LY96	9,8	RNU4ATAC	44,5	CCL3L1/CCL3L3	4,7
	HMOX1	8,3	CCL3L1/CCL3L3	6,0	HMOX1	6,3	HIST1H4A	44,2	JUN	4,7
	HIST1H4A	5,9	HMOX1	5,8	EMP1	5,5	mir-320	41,4	EMP1	4,6
	mir-320	5,8	MLLT11	5,7	HIST1H4A	5,5	RNU12	35,4	VIM	4,6
	JUN	5,8	EMP1	5,2	JUN	5,1	HIST1H2BB	30,2	MLLT11	4,4
	ALB	5,6	GABARAPL1	5,2	CCL3L1/CCL3L3	4,6	VTRNA1–1	22,5	GABARAPL1	4,3
	GABARAPL1	5,4	RNU11	5,0	UPP1	4,6	HIST1H2AG	22,2	HMOX1	4,1
	RNU12	5,2	CYP1A1	4,7	CYP1A1	4,2	ALB	22,0	ANXA1	3,8
Top down-regulated genes	MYC	−5,4	MYC	−10,3	MYC	−10,4	MYC	−9,9	MYC	−8,3
	MYB	−4,5	MS4A3	−7,0	MS4A3	−6,8	PAFAH1B3	−8,5	MS4A3	−8,3
	LRP3	−4,2	MYB	−5,7	CCND2	−5,2	LRP3	−6,8	CTSG	−4,9
	PPIA	−3,9	CTSG	−5,2	MYB	−5,2	PPIA	−6,7	MT1G	−4,4
	MS4A3	−3,6	CCND2	−4,8	CTSG	−4,5	MYB	−6,0	CCND2	−4,4
	DTD1	−3,5	MT1G	−4,5	RPL29	−4,3	NIP7	−5,9	RPL29	−4,3
	IFITM2	−3,5	IFITM2	−4,2	RNASE3	−3,8	DTD1	−5,9	MYB	−3,9
	WASH1	−3,5	RPL29	−4,2	IFITM2	−3,8	CDCA7	−5,4	NOP16	−3,3
	PAFAH1B3	−3,4	ELANE	−3,8	RRS1	−3,4	DUT	−5,4	RPL36A	−3,3
	CCND2	−3,3	LRP3	−3,6	RPS15	−3,3	MS4A3	−5,3	RRS1	−3,3

**Table 2:** Comparison of microarray gene expression profiling and real-time RT-PCR for six selected genes

Genes	Fold change									
	Shikonin		Isobutyrylshikonin		β,β-Dimethylacrylshikonin		Isovalerylshikonin		2-Methylbutyrylshikonin	
	Microarray	RT-PCR	Microarray	RT-PCR	Microarray	RT-PCR	Microarray	RT-PCR	Microarray	RT-PCR
**JUN**	5.8	30.67	7.1	160.45	5.1	19.37	7.7	37.24	4.7	32.05
**GABARAPL1**	5.4	78.16	5.2	78.14	4.1	56.77	4.5	96.57	4.3	112.43
**LY96**	9	69.84	12.4	161.44	9.8	49.32	4.7	93.54	11.2	103.92
**IFITM2**	−3.5	−1.05	−4.2	−1.31	−3.8	−1.59	−5.1	−1.27	−3.2	−1.32
**MYC**	−5.4	−2.37	−10.3	−2.30	−10.4	−5.97	−9.9	−5.26	−8.3	−2.20
**MS4AS**	−3.6	−1.72	−7.0	−2.62	−6.8	−10.03	−5.3	−4.63	−8.3	–1.24
**R-value**	0.9011		0.9386		0.8662		0.7954		0.8480	

The correlation coefficient (R-value) between mRNA expression values determined by microarray hybridization and real-time RT-PCR were caculated with Pearson correlation test

We subjected all data obtained by microarray analyses and subjected them to Ingenuity Pathway Analysis (IPA). The deregulated genes were correlated with several molecular and cellular functions, including cell death and survival, cellular growth and proliferation, cellular development, cellular movement, gene expression, cell cycle, cell-to-cell signaling and interaction, etc. Figure 3B and 3C display the top cellular functions and pathways affected by shikonin and derivatives in U937 cells.

Furthermore, an upstream regulator analysis was performed with IPA to identify transcriptional regulators, kinases, or enzymes that may be responsible for gene expression changes in U937 cells after treatment. Table 3 shows the upstream regulators predicted by IPA to be activated or inhibited by shikonin or derivatives. The most likely activated and inhibited upstream regulators for each compound were underlined. MYC was found to be a commonly inhibited transcription regulator by shikonin and derivatives. Figure 3D shows the deregulated genes controlled by MYC.

**Table 3:** Upstream regulators presumably affected by shikonin and its derivatives after 24 h in U937 cells

Shikonin		Isobutyrylshikonin		β,β-Dimethylacrylshikonin		Isovalerylshikonin		2-Methylbutyrylshikonin	
Activated	Inhibited	Activated	Inhibited	Activated	Inhibited	Activated	Inhibited	Activated	Inhibited
TP53	MYC	PDGF BB	MYC	PDGF BB	MYC	FOXO3	MYC	PDGF BB	MYC
ATF4	BRD4	ATF4	BRD4	CEBPA	BRD4	NANOG	BRD4	TNF	BRD4
ATF2	MAX	FOXO3	Mek	TREM1	Hdac	SMAD4		TCR	Mek
CDKN2A	TRIB3	ELANE	MGEA5	FOXO3	Mek	RUVBL1		TGM2	mir-146
FOXL2	MNT	TREM1		TREM1	MGEA5	PAF1		EGF	miR-155–5p
CEBPB	Mek	CEBPB		TP53		HIF1A		PF4	TAB1
PGR		CDKN2A		TGM2		PPRC1		TREM1	MGEA5
IGF1		FOXL2		CDKN2A				CCL5	
PPRC1		TP53		CEBPB				KRAS	
FOXO1		TGM2		FOXO1				Fcer1	
		ERK1/2		GLI1				TGFB1	
		IGF1		SELPLG				IKBKB	
		FOXO1		NEDD9				NFkB	
		NFkB		EP300				IRF3	
		EP300		Jnk				CEBPB	
		SELPLG		Cg				Jnk	
		PPRC1						SELPLG	
		Jnk						IGF1	
		CXCL12						CAMP	
		NEDD9							
		NEDD9							

The most likely activated and inhibited upstream regulators for each compound were underlined

### Inhibition of c-MYC expression and its DNA-binding activity by shikonin and derivatives

**Figure 4:**
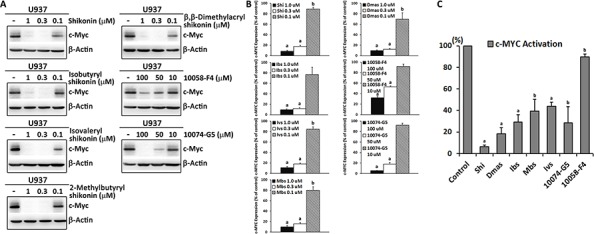
Inhibition of c-MYC protein expression and DNA-binding activity by shikonin, its derivatives, 10058-F4 and 10074-G5 in U937 cells **A.** Western blot analysis of c-MYC expression after 24 h treatment with these compounds. β-Actin was used as loading control. **B.** Digitalized graphs of c-MYC protein levels as quantified by FluorChem Q. Data were normalized to β-actin expression and represented as means ± SEM of three independent experiments (*n* = 3). ^a^, *p* < 0.01 *vs.* control; ^b^, *p* < 0.05 *vs.* control, calculated by two-tailed Student's *t* test. **C.** Determination of DNA binding activity of c-MYC by Trans-AM ELISA-based kit. Nuclear extracts were obtained after treatment of U937 cells with 0.3 μM shikonin and its derivatives or 50 μM 10058-F4 and 10074-G5 for 24 h. Protein/oligonucleotide binding activity was measured by colorimetric analysis with 10 μg of nuclear extracts. The absorbance at 450 nm was recorded by an ELISA plate reader. Results are presented as percentage with respect to the untreated control and represented as mean values ± SEM of three independent experiments (*n* = 3). ^a^, *p* < 0.01 *vs.* control; ^b^, *p* < 0.05 *vs.* control, calculated by two-tailed Student's *t* test. Shi, shikonin; Ibs, isobutyrylshikonin; Dmas, β,β-dimethylacrylshikonin; Ivs, isovalerylshikonin; Mbs, 2-methylbutyrylshikonin.

The microarray analysis indicated that *MYC* was not only the most commonly down-regulated gene, but also the common upstream regulator affected by shikonin and its four derivatives. Moreover, cell cycle G1/S check point regulation, which is mainly regulated by MYC [6], was also the top canonical pathway affected by all shikonins. Therefore, we supposed that MYC itself may be a potential target of this type of compounds. To analyze this hypothesis, we performed Western blotting to prove whether shikonin and its derivatives affect c-MYC expression. Two known c-MYC inhibitors, 10074-G5 and 10058-F4 were used as control drugs. As shown in Figure 4A, 4B, shikonin and all four derivatives indeed revealed a strong inhibition of c-MYC expression in U937 cells at 1 μM and 0.3 μM. This effect was greater than that of 10058-F4 and comparable to that of 10074-G5, however at much lower concentrations than the control compounds.

Next, we attempted to determine, whether the inhibition of c-MYC expression in U937 cells by shikonin and its derivatives was associated with decreased transcriptional activity and DNA binding activity of c-MYC. For this purpose, we used a specific ELISA-based DNA-binding assay. In Figure 4C, the DNA binding activity of c-MYC in nuclear extracts of U937 cells was suppressed to different extents by treatment of 0.3 μM shikonin and derivatives or 50 μM of the two control inhibitors. These results were in concordance with the Western blot analysis, since 10058-F4 caused the weakest inhibition, while shikonin and derivatives showed strong inhibition, which was similar to 10074-G5. This clearly suggests that shikonin and its derivatives possess MYC inhibitory activities.

### Involvement of AKT and ERK1/2, JNK MAPK signaling in shikonin-induced c-MYC down-regulation

**Figure 5:**
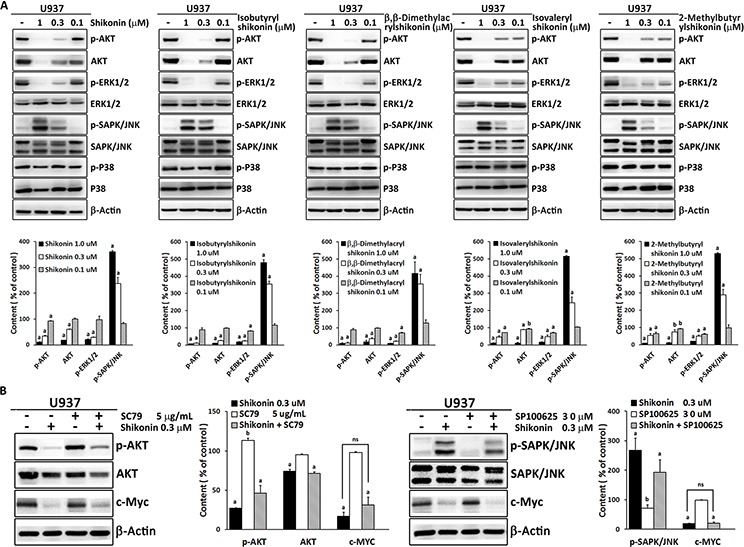
**A.** Effect of shikonin and its derivatives on AKT/MAPK signaling in U937 cells as determined by Western blotting. Band density of phosphorylation of AKT, ERK1/2, SAPK/JNK and P38 was normalized to their corresponding total protein levels. β-actin was used as loading control. Below the Western blots, the corresponding digitalized graphs of affected proteins levels for each compound are shown. Data are represented as mean values ± SEM of three independent experiments (*n* = 3). ^a^, *p* < 0.01 *vs.* control; ^b^, *p* < 0.05 *vs.* control, calculated by two-tailed Student's *t* test. **B.** Western blot analysis of the indicated proteins in U937 cells treated with 0.3 μM shikonin in the absence or presence of 5 μg/mL SC79 or the absence or presence of 30 μM SP600125 for 24 h. Digitalized graphs of affected proteins levels are shown aside. Data are represented as mean values ± SEM of three independent experiments (*n* = 3). a, *p* < 0.01 *vs.* control; b, *p* < 0.05 *vs.* control; ns, no significance, calculated by two-tailed Student's *t* test.

It is known that c-MYC is regulated by multiple signaling pathways, including MAPK and AKT signal transduction cascades [18, 26–28]. Therefore, we further employed Western blot analysis to evaluate, whether the MAPK and AKT signal transduction pathways were involved in shikonin-induced c-MYC down-regulation (Figure 5A). The results showed that shikonin inhibited phospho-ERK1/2 and activated phospho-SAPK/JNK without influencing total ERK1/2 and SAPK/JNK expression. However, both phospho-AKT and total AKT expression were reduced by shikonin. No appreciable changes were detected in phospho-p38 or total p38. Comparable results were also found for the other shikonin derivatives.

Then, we examined which signaling pathway may play a critical role in regulation of c-MYC. A specific AKT activator (SC79) and a specific JNK inhibitor (SP600125) were independently used in combination with shikonin and the expression of c-MYC was measured (Figure 5B). Western blotting analysis showed neither SC79 nor SP600125 appreciably reversed shikonin-induced c-MYC reduction, though they partly attenuated shikonin's effect on phospho-AKT and phospho-SAPK/JNK, indicating that the down-regulation of c-MYC probably resulted from the joint contributions of AKT and ERK1/2, SAPK/JNK MAPK signaling cascades and the direct binding of shikonin to c-MYC.

### Inhibition of c-MYC with involvement of AKT and MAPK signaling cascades is a general mechanism for shikonin

**Figure 6:**
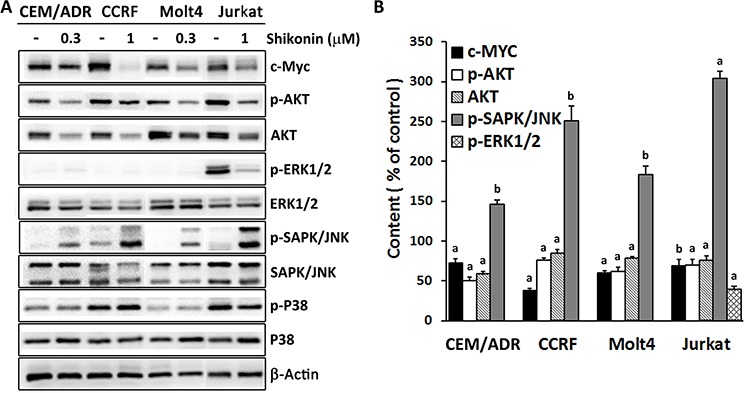
**A.** Effect of shikonin on c-MYC expression and AKT/MAPK signaling in four different leukemia cell lines as determined by Western blotting. CEM/ADR5000, CCRF-CEM, Molt4 and Jurkat cells were treated with 0.3 or 1 μM shikonin depending on their sensitivities to shikonin treatment for 24 h and the whole cell lysates were subjected to Western blotting. Band density of phosphorylation of AKT, ERK1/2, SAPK/JNK and P38 was normalized to their corresponding total protein levels. Others were normalized to β-actin, which was also used as loading control. **B.** The digitalized graphs of affected protein levels were quantified by FluorChem Q for each cell line. Data are represented as mean values ± SEM of three independent experiments (*n* = 3). ^a^, *p* < 0.01 *vs.* control; ^b^, *p* < 0.05 *vs.* control, calculated by two-tailed Student's *t* test. CEM/ADR, CEM/ADR5000; CCRF, CCRF-CEM.

In addition to U937 cells, we investigated four other different leukemia cell lines (CEM/ADR5000, CCRF-CEM, Molt4 and Jurkat) to prove, whether or not inhibition of c-MYC expression is a general mechanism for shikonin in killing leukemia cells. We first tested the sensitivities of four cell lines to shikonin. The IC_50_ values after 24 h for CEM/ADR5000, CCRF-CEM, Molt4 and Jurkat cells were 0.29 ± 0.03 μM, 1.68 ± 0.23 μM, 0.24 ± 0.03 μM and 0.97 ± 0.14 μM, respectively. Then, the cells were treated with 0.3 or 1 μM shikonin for 24 h depended on their different sensitivities, followed by whole cell lysate extraction for Western blot analyses. The effect of shikonin on c-MYC expression as well as AKT and MAPK signaling cascades were measured. As displayed in Figure 6A, shikonin suppressed c-MYC expression to a different extent in all tested cell lines. Meanwhile, reduction of phospho-AKT and total AKT, activation of phospho-SAPK/JNK were also found in all four cell lines upon shikonin treatment. Phospho-ERK1/2 was only activated in Jurkat cells and was inhibited by shikonin. There were still no significant changes in phospo-p38 and total p38 in all cell lines. All these results were comparable to those of U937 cells, suggesting that AKT, ERK1/2, SAPK/JNK/MAPK signaling cascades involved c-MYC inhibition play a general role in shikonin-caused leukemia cell death.

### Molecular docking

**Figure 7:**
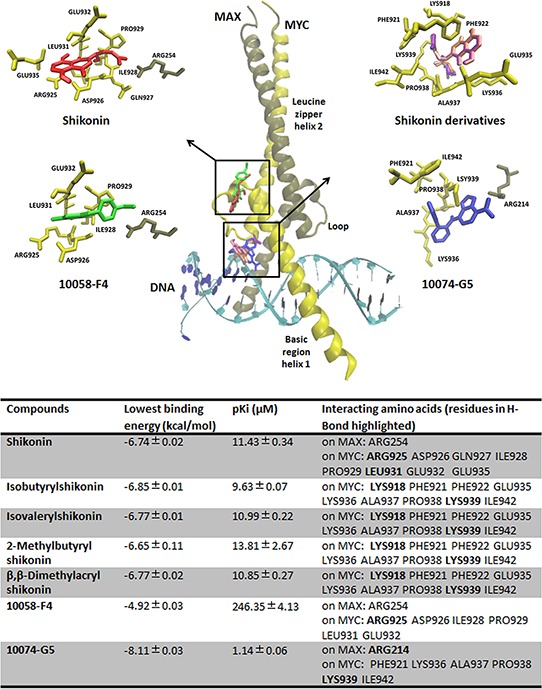
Docking modes and binding energies of shikonin, its derivatives, 10058-F4 and 10074-G5 on MYC/MAX complex (PDB code: 1NKP) The proteins were represented in yellow (MYC) and brown (MAX) in New Cartoon format, while the chemical molecules were represented in different colors. Shikonin preferentially bound to the same pharmacophore as the control drug 10058-F4, while the other derivatives docked to the same domain as the second control drug 10074-G5. Both binding sites were in close proximity to the DNA binding region of MYC/MAX complex.

To further investigate the possible interaction of shikonin and derivatives with c-MYC, molecular *in silico* docking studies were performed. There exist at least two binding sites in the basic helix-loop-helix (bHLH) leucine zipper domain of the MYC-MAX complex [16]. Our blind docking results showed that shikonin preferentially bound to the same site as the control drug 10058-F4, while the other derivatives docked to the domain, where the other control drug 10074-G5 bound. Both binding sites were in close proximity to the DNA binding region of MYC and MAX. Shikonin and derivatives formed hydrogen bonds with residues at the DNA binding regions of MYC, *i.e*. Arg925, Lys939. The lowest binding energies of shikonin and its derivatives were similar and in a range from −6.65 ± 0.11 kcal/mol to −6.85 ± 0.01 kcal/mol. These values were also comparable with those of the control inhibitors, indicating the feasibility for shikonin and its derivatives directly targeting the c-MYC complex and inducing MYC-related gene expression changes. Docking modes and the binding energies for shikonin and its derivatives and the control inhibitors on the MYC-MAX complex are summarized in Figure 7.

## DISCUSSION

In the present investigation, we showed that shikonin and 14 derivatives revealed strong cytotoxicity towards U937 leukemia cells. Especially, the four derivatives–isobutyrylshikonin, 2-methylbutyrylshikonin, isovalerylshikonin and β,β-dimethylacrylshikonin–that were more effective than the lead compound shikonin. Moreover, it was intriguing to observe that shikonin and its homochiral derivatives were more active than their enantiomers. For example, the IC_50_ value for shikonin was 25 folds lower than that of alkannin. Similarly, isobutyrylshikonin, 2-methylbutyrylshikonin and β-hydroxyisovalerylshikonin also showed lower IC_50_ values than their corresponding enantiomers, isobutyrylalkannin, 2-methylbutyrylalkannin and β-hydroxyisovalerylalkannin, indicating that differences in the stereochemistry at C-11 may influence the activity. This structure-activity relationship was consistent with what we found in glioblastoma cell lines and other reports [24, 29]. Another interesting point is that leukemia cell lines were more sensitive than solid tumor cell lines [25]. A plethora of studies, including clinical research, reported that leukemia is generally very sensitive to anticancer reagents that either block the cell cycle process or cause apoptosis [30–33]. Therefore, we proposed that the particular activities of shikonin and its derivatives against leukemia cells may be correlated with their strong effect to induce cell cycle disruption and apoptosis. Previous studies reported that shikonin caused an arrest of U937 cells in the G1 and S phase and decreased expression of cell cycle-related proteins, such as cyclin D, CDK and PCNA [25, 34]. However, the mechanism of this effect remains unknown as of yet. By means of microarray-based gene expression analysis, we noticed that the transcription factor MYC, which plays a very critical role in cell cycle control, was the commonly deregulated molecule by shikonin and its derivatives. MYC can influence cell cycle progression through several parallel mechanisms, such as transcription of target genes including CDKs, cyclins and E2F that encode many important positive cell cycle regulators, hyperactivating cyclin/Cdk complexes through the induction of CDK-activating kinase (CAK) and CDC25 phosphatases as well as impairment of cell cycle inhibitors such as p21 and p27 [35]. Inhibition of MYC expression, down-regulation or inactivation of MYC in cycling cells results in cell cycle arrest and impairs cell cycle progression in various cell lines, including human lymphoid and myeloid cells [35–37]. GO enrichment analysis by DAVID showed that *MYC* was indeed widely involved in the main biological processes such as cell cycle and cell death. Additionally, cellular pathways analysis by IPA also provided supporting information that G1/S checkpoint regulation was the most disturbed pathway upon treatment with shikonin and its derivatives. This further indicated that cell cycle arrest in U937 cells resulted from deregulation of MYC and shikonin and its derivatives may be potential MYC inhibitors. To prove this hypothesis, we examined the effect on c-MYC expression and its transcriptional activity by Western blotting and a specific ELISA-based DNA-binding assay. Shikonin and its derivatives exerted remarkable inhibition on c-MYC expression and its DNA-binding activity, which was much better than the known MYC inhibitor 10058-F4 and comparable to 10074-G5. Moreover, the effective concentrations for c-MYC inhibition were much lower than those of both control drugs. To the best of our knowledge, this is the first report for shikonin to kill cancer cells by targeting c-MYC, suggesting a novel role of shikonins as antitumor agents. In addition to *MYC*, another two cancer-related molecules *MYB* and *MS4A3* were also significantly down-regulated by all five shikonin derivatives. *MYB* is a proto-oncogene which is overexpressed in most human myeloid and acute lymphoid leukemias [38]. It actively contributes to leukemogenesis by promoting proliferation, suppressing apoptosis and blocking differentiation [39]. *MS4A3* (also known as *HTm4*) encodes a member of membrane-spanning 4-domains subfamily, which acts as an important cell cycle regulator in various cancers, especially in hematological malignancies [40, 41]. Therefore, it was likely that the dysregulation of *MYB* and *MS4A3* also contribute to shikonin and its derivatives induced cell cycle arrest and cell death in U937 cells. It is worth performing further experiments to clarify the potential effect of shikonin on them.

On the other hand, we clarified the death mode of U937 cells by annexin V-PI double staining in the presence or absence of the specific necroptosis inhibitor Nec-1 and the caspase inhibitor z-VAD-fmk. IC_50_ concentrations of shikonin and derivatives mainly induced cell death by caspase-dependent apoptosis, as evidenced by a remarkable decrease in annexin V+/PI− and annexin V+/PI+ cells in presence of z-VAD-fmk. This was in agreement with previous reports that low concentrations of shikonin induced caspase-dependent apoptosis in mitochondriae in leukemia cells [42]. However, in addition to apoptosis, we found that necroptosis seemed to be also induced by shikonin and derivatives, as Nec-1 could partly rescued cell from death. This means that necroptosis, which was induced mostly by high dose of shikonin or its derivatives [42–44], perhaps could also be triggered by low concentrations of shikonins as a secondary death mechanism. Additional experiments such as electron microscopy analysis will help to further confirm this effect. The role of c-MYC in apoptosis is intricate, depended on the specific cell type and the physiological status of the cell. Both reduction and overexpression of c-MYC can lead to apoptosis [45, 46]. However, in hematopoietic cells, apoptosis is closely correlated with reduction of c-MYC expression. For example, apoptosis of CEM lymphoblastoid cells induced by oxysterol 25-hydroxycholesterol was preceded by ≥ 90% reduction in c-MYC levels [47]. Treatment of K562 erythroleukemia cells with the protein phosphatase inhibitors okadaic acid or calyculin A caused down-regulation of c-MYC and MAX expression and led to apoptosis [48]. The myeloid HL-60 leukemia cell line also underwent apoptosis by treatments that reduce c-MYC expression [49]. In addition, the small molecule c-MYC inhibitor 10058-F4 inhibited proliferation and induced apoptosis through the mitochondrial pathway of apoptosis in several acute myelocytic leukemia cell lines [50]. Therefore, we believe that apoptosis induced by shikonin and its derivatives in U937 cells is also associated with inhibition of c-MYC expression.

In an attempt to explore, how shikonin and its derivatives inhibit c-MYC expression and activity, we examined the effect of shikonins on signaling pathways that may regulate c-MYC expression. It has been showed that shikonin or its derivatives inhibit cancer cells via AKT/mTOR and MAPK signaling cascades [51–55]. Our results are consistent with these previous findings, as shikonin and derivatives demonstrated significant effects on ERK1/2, SAPK/JNK MAPK kinases pathways and AKT pathways. These pathways play an important role in the control of c-MYC protein stability, accumulation and subsequent transcriptional activity. c-MYC protein stability is strongly influenced by phosphorylation of two adjacent N-terminal sites, threonine 58 (Thr58) and serine 62 (Ser62), which display opposing roles. Phosphorylation of Ser62 that is mediated by ERK pathway kinase activity stabilizes c-MYC, while phosphorylation of Thr58 by GSK-3 promotes c-MYC degradation [56]. GSK-3 activity is usually inhibited through PI3K/AKT. Only if AKT activity declines, GSK-3 has the capacity to phosphorylate Thr58 and to induce the degradation of c-MYC [57]. Meanwhile, JNK also contributes to c-MYC stability by increasing its ubiquitin-dependent degradation via a δ-like domain [58]. In addition to the influence on c-MYC protein stability, the PI3K/AKT and MAPK pathways also take part in the regulation of c-MYC-mediated transcription by phosphorylating and promoting MAD1 degradation. MAD1 suppresses c-MYC transcriptional activity by competing with c-MYC for heterodimerization with its partner MAX [26]. Thus, inhibition of the ERK/PI3K/AKT pathway, or activation of JNK signaling may lead to down-regulation of c-MYC. Our results showed that reduction of c-MYC expression by shikonins and its derivatives was closely correlated with inhibition of phosphorylation of ERK1/2 and AKT and activation of phosphorylation of SAPK/JNK. This further confirms the role of these pathways for c-MYC regulation. However, neither the inhibition of AKT nor the activation of SAPK/JNK alone appreciably reversed shikonin-induced c-MYC suppression. This indicates a comprehensive effect of ERK1/2, JNK MAPK and AKT signaling for down-regulation of c-MYC and a direct interaction of shikonin with c-MYC.

Our molecular docking studies demonstrated the binding of shikonin and its derivatives to the DNA-binding domain of c-MYC in a similar manner as the known c-MYC inhibitors 10074-G5 and 10058-F4. Additionally, c-MYC deregulation may in turn also act on AKT activity. Recent studies reported that reduced c-MYC levels led to decreased AKT activity *in vitro* and *in vivo* [59, 60]. Thus, AKT down-regulation upon shikonin treatment may be further reinforced as negative feedback of c-MYC down-regulation. Co-targeting of AKT and c-MYC has been recently shown to be a synergistic treatment strategy for leukemia therapy [61, 62], since shikonin and its derivatives strongly deregulate the AKT signaling pathway and directly inhibit c-MYC activity. Therefore, they represent promising candidates for leukemia treatment.

Moreover, the mechanism of shikonin in U937 cells also applies for other leukemia cell lines, including the multidrug-resistant cell line CEM/ADR5000. This indicates that inhibition of c-MYC with involvement of the ERK/JNK/MAPK and AKT pathways represents a general mechanism for shikonin and its derivatives in killing leukemia cells. Notably, multidrug-resistant CEM/ADR5000 cells were even more sensitive to shikonin than the wild-type cell line CCRF-CEM, as evidenced by the lower IC_50_ value. The phenomenon of hypersensitivity of multidrug-resistant cells has been termed collateral sensitivity [63]. Considering the important role of c-MYC in drug-resistant leukemia, we assume that shikonin's collateral sensitivity in CEM/ADR5000 cells may be also c-MYC-related. This opens avenues for shikonin and its derivatives for combination therapies to treat otherwise drug-resistant tumors.

In a word, the novel mechanisms for shikonin and its derivatives reported in the present study make these compounds attractive candidates for the treatment of hematological malignancies.

## MATERIALS AND METHODS

### Chemicals

Shikonin and derivatives were isolated and purified from *Arnebia euchroma* and *Onosma paniculata* as described [22, 23]. The chemical structures are shown in Figure 1A. Stock solutions (50 mM) were prepared in DMSO, stored at − 20°C and diluted to the final concentration in fresh media before each experiment. SC79, 10058-F4 and 10074-G5 were purchased from Sigma-Aldrich (Taufkirchen, Germany) and SP100625 was purchased fom Enzo Life Sciences (Lörrach, Germany).

### Cell cultures

The parental human monocytic AML cell line U937, ALL cell lines Molt4 and Jurkat were obtained from the German Cancer Research Center (DKFZ, Heidelberg, Germany). The original source of the cell lines is the American Type Culture Collection (ATCC, USA). ALL cell lines CCRF-CEM and its derived CEM/ADR5000 cell lines were generously provided by Prof. Axel Sauerbrey (Department of Pediatrics, University of Jena, Jena, Germany). Cells were cultivated in complete RPMI 1640 medium with 2 mM L-glutamine (Invitrogen, Darmstadt, Germany) supplemented with 10% fetal bovine serum and 1% of a stock solution of 10,000 U/mL penicillin G and 10 mg/mL streptomycin at 37°C in a humidified air incubator (95%) containing 5% CO_2_. CEM/ADR5000 cells were continuously treated with 5000 ng/mL doxorubicin to maintain the multidrug-resistance phenotype. All experiments were performed with cells in the logarithmic growth phase.

### Cell viability assay

Cell viability was evaluated by the resazurin assay. This test is based on the reduction of the indicator dye, resazurin, to the highly fluorescent resorufin by viable cells. Nonviable cells rapidly lose the metabolic capacity to reduce resazurin and, thus, do not produce a fluorescent signal. In brief, 2 × 10^4^ cells were sowed in a 96-well culture plate in a total volume of 100 μL for each well. Marginal wells were filled with 200 μL of pure medium, in order to minimize the effects of evaporation. Besides, wells filled with medium served as negative control to determine background fluorescence that may be present. Then, cells were immediately treated with different concentrations of shikonin and its derivatives. After 24 h or 72 h, 20 μL resazurin (Sigma-Aldrich, 0.01% w/v in ddH_2_O) was added to each well and the plates were incubated at 37°C for 4 h. Fluorescence was measured in an Infinite M2000 Proplate reader (Tecan, Crailsheim, Germany) using an excitation wavelength of 544 nm and an emission wavelength of 590 nm. Each assay was done at least three times, with six replicates each. The cytotoxic effect of the treatment was determined as percentage of viability and compared to untreated cells. The toxicity of compounds was determined by means of the formula:

$$\text{Cell Viability }(\%\text{ of control})=\frac{\text{absorption from sample well }-\text{ absorption from medium}}{\text{absorption from solvent treated cells}-\text{absorption from medium}}\times 100$$

The calculated cell viability (y-axis) was plotted against the log drug concentration (x-axis) using Microsoft Excel. The obtained curve was used to determine the IC_50_ value, which represented the concentration of the test compound required to inhibit 50% of cell proliferation.

### Assessment of cell death by flow cytometry

The cell death mode induced by shikonin and its derivatives was analyzed by annexin V-PI double staining. Annexin V is an intracellular protein that calcium-dependently binds to phosphatidylserine (PS), which translocates from the intracellular leaflet of the plasma membrane to the external leaflet during early apoptosis. Propidium iodide (PI) is excluded by living or early apoptotic cells with intact membranes and stains late apoptotic and necrotic cells with red fluorescence due to DNA intercalation. Therefore, cells with annexin V (−) and PI (−) are considered to be alive, while cells with annexin V (+) and PI (−) are in early apoptosis. Cells in late apoptosis or necrosis are both annexin V and PI positive. Briefly, 5 × 10^5^ U937 cells were treated 50 μM necrostatin-1 (Nec-1; Enzo Life Sciences) or 50 μM z-VAD-fmk (Selleckchem, Munich, Germany) 1 h prior to co-incubation with IC_50_ concentrations of shikonin or its derivatives for 24 h. Following incubation, cells were collected and incubated with annexin V and PI staining solution (BioVision, Heidelberg, Germany) according to the manufacturer's protocol. Subsequently, cells were measured with FACS Calibur analyzer (Becton-Dickinson Biosciences). For each sample, 2 × 10^4^ cells were counted. The annexin V-FITC signal was measured with 488 nm excitation and detected using a 530/30 nm band pass filter. The PI signal was analyzed with 561 nm excitation and detected using a 610/20 nm band pass filter. All parameters were plotted on a logarithmic scale. Cytographs were analyzed using FlowJo software (Celeza, Olten, Switzerland).

### Microarray gene expression profiling

U937 cells were treated with shikonin, isobutyrylshikonin, β,β-dimethylacrylshikonin, isovalerylshikonin and 2-methylbutyrylshikonin, at IC_50_ concentrations or DMSO as solvent control for 24 h, before total RNA was isolated using InviTrap spin Universal RNA Mini kit (250) (STRATEC Molecular, Berlin, Germany) according to the manufacture's instruction. RNA concentrations were determined using the nanodrop spectrophotometer (Nanodrop Technologies, Thermo Fisher, Dreieich, Germany). Microarray hybridizations were performed in duplicates for treated samples and for control samples by the Genomics and Proteomics Core Facility at the German Cancer Research Center (DKFZ, Heidelberg). Briefly, 1 μg total RNA was used for complementary DNA (cDNA) synthesis, followed by an amplification/labeling step (*in vitro* transcription) to synthesize biotin-labeled cRNA according to the MessageAmp II aRNA Amplification kit (Ambion, Inc., Austin, TX, USA). Biotin-labeled cRNA samples for hybridization on Illumina Human HT-12 BeadChip arrays were prepared according to Illumina's recommended sample labeling procedure based on the modified Eberwine protocol [64]. The cRNA was column purified according to TotalPrep™ RNA Amplification Kit (Life Technologies, Darmstadt, Germany) and eluted in 60–80 μL water. Hybridization was performed according to the manufacturer's instructions. Microarray scanning was done using an Illumina^®^ BeadStation array scanner (Illumina, San Diego, CA, USA), setting adjusted to a scaling factor of 1 and PMT settings at 430. Data was extracted for each individually, and outliers were removed, if the median absolute deviation (MAD) exceeded 2.5. Then, mean average signals and standard deviations were calculated for each probe. Data analysis was done by using the quantile normalization algorithm without background subtraction, and differentially regulated genes were defined by calculating the standard deviation differences of a given probe in a one-by-one comparison of samples or groups. The data obtained was further filtered with Chipster software including the steps that filtering of genes by two times standard deviation and a subsequent assessment of significance using empirical Bayes *t*-test (*p* < 0.05) with Bonferroni correction. Filtered genes were fed into Ingenuity Pathway Analysis software (IPA; Ingenuity Systems, Redwood City, CA, USA) and analyzed by the core analysis tool to determine cellular networks and functions affected by each drug treatment. The results of the core analyses were further studied using the comparison analysis tool, offering the possibility to compare datasets of samples treated by different compounds. Gene ontology (GO) enrichment analysis was performed using online Database for Annotation, Visualization and Integration Discovery (DAVID) programme (https://david.ncifcrf.gov/) to investigate functional categorization of common genes [65, 66].

### Real-time reverse transcription-PCR

Real-time RT-PCR was performed with the same samples used for microarray experiments. Total RNA samples were converted to cDNA with random hexamer primers by RevertAid H Minus First Strand cDNA Synthesis Kit (Thermo Scientific, Waltham, MA). Oligonucleotides were synthesized by Eurofins MWG Operon (Ebersberg, Germany). The efficiency of all primer pairs used for real-time PCR expression was better than 90%. Quantification of cDNA was performed on CFX384 Real-Time PCR Detection System (Bio-Rad) using a Hot Start Taq EvaGreen qPCR Mix (Axon). RT-PCR was performed with an initial denaturation at 95°C for 10 min followed by 40 cycles including strand separation at 95°C for 15 s, annealing at 57.4°C for 40 s and extension at 72°C for 1 min. After PCR product amplification, melting curves were computed. Expression levels were normalized to the transcription level of the housekeeping gene *RPS13*. All samples were run in duplicates and the experiment was repeated once.

### Preparation of protein lysates and western blotting

Cells were washed twice with PBS after treatment with the indicated concentrations of shikonin, its derivatives, 10074-G5 or 10058-F4 for 24 h and lysed in lysis buffer (M-PER Mammalian Protein Extraction Reagent, Thermo Scientific, plus protease inhibitor, Roche, Mannheim, Germany) containing phosphatase inhibitor (Roche). After shaking 30 min at 4°C, the lysate was centrifuged at 14,000 × g for 15 min and the supernatant was quantified by Nanodrop. Equal amounts of protein extracts (30 μg) were separated by 10% SDS-PAGE and electroblotted onto a PVDF membrane (Millipore, Darmstadt, Germany). The membrane was first rinsed with TBST (20 mM Tris-HCl, pH 7.4, 0.15 M NaCl, 0.1% Tween 20) and then blocked with 5% (w/v) bovine serum albumin in TBST for 1 h at room temperature. The blocked membrane was subsequently incubated overnight at 4°C with specific primary antibodies (Cell Signaling, Technology/New England Biolabs, Frankfurt, Germany). After washed three times with TBST for 10 min, the membrane was incubated for 1 h at room temperature with HRP-conjugated secondary antibody (Cell Signaling). After the membrane had been washed with TBST, the immunoreactivity was revealed by use of a Luminata Classico Western HRP Substrate (Millipore), and the densities of the protein bands were quantified by FluorChem Q software (Biozym Scientific Company, Oldendorf, Germany). β-Actin was used as loading control.

### DNA binding activity of c-MYC transcription factors

The c-MYC DNA binding activity assays were performed using TransAM enzyme-linked Immunosorbent assay (ELISA)-based kits (Active Motif, Rixensart, Belgium) according to the manufacturer's protocol. Briefly, 10 μg of nuclear extracts from control, shikonin, derivatives, 10074-G5 or 10058-F4-treated cells were separately incubated in a 96-well plate immobilized with an oligonucleotide containing the c-MYC consensus binding site (5′-CACGTG-3′). The active forms of transcription factors from extracts, which specifically bound to this oligonucleotide, were detected by a primary antibody against c-MYC in an ELISA-like format. The absorbance of the sensitive colorimetric reaction mediated by a secondary HRP-conjugated antibody was measured on the Infinite M2000 Proplate reader (Tecan) at 450 nm with a reference wavelength of 655 nm.

### Molecular docking

The X-ray crystallography based structure of MYC/MAX complex (PDB code: 1NKP) was obtained from RCSB Protein Data Bank (http://www.rcsb.org/pdb/home/home.do) and used as docking template throughout the docking calculations. The bHLH and leucine zipper regions of the proteins were covered in the PDB file. The 2D structures of shikonin and its derivatives were energy-minimized and converted to 3D structures compatible for docking operation using the open source program Corina (Molecular Networks, Erlangen, Germany). Two known MYC inhibitors, 10074-G5 and 10058-F4, were used as control drugs to compare their binding modes and affinities with shikonin and its derivatives [17, 67]. Molecular docking was then carried out with Autodock program (AutoDock 4.2, The Scripps Research Institute, La Jolla, CA, USA) following a protocol previously reported by us [68]. Docking parameters were set to 250 runs and 25,000,000 energy evaluations for each cycle. VMD (Visual Molecular Dynamics) was used as visualization tool to further get a deeper insight on the binding modes obtained from docking.

## SUPPLEMENTARY TABLE


