# Supplementary material for: Inhibition of c-MYC with involvement of ERK/JNK/MAPK and AKT pathways as a novel mechanism for shikonin and its derivatives in killing leukemia cells
Source: Oncotarget. 2015 Oct 9;6(36):38934–51. doi: 10.18632/oncotarget.5380 (PMC4770748; doi:10.18632/oncotarget.5380)
Supplement: Supplementary file 1 [file oncotarget-06-38934-s001.pdf]

SUPPLEMENTARY TABLE

Supplementary Table S1: Go enrichment analysis of 91 cmmonly deregulated genes by shikonin and its derivatives after 24 h in U937 cells

Go category	Go term	Gene count	*P*-value	Gene involved
Biological process	cell cycle process	10	6.01E-03	NOLC1, CCND2, RASSF1, PSMC1, RRS1, RUVBL1, DYNC1H1, PPP1R15A, GADD45A, MYC
	cell death	10	2.57E-02	IER3, AIMP2, HMOX1, JUN, TNFAIP8, LGALS12, PPP1R15A, GADD45A, MYC, DDIT4
	death	10	2.67E-02	IER3, AIMP2, HMOX1, JUN, TNFAIP8, LGALS12, PPP1R15A, GADD45A, MYC, DDIT4
	cell cycle	10	3.91E-02	NOLC1, CCND2, RASSF1, PSMC1, RRS1, RUVBL1, DYNC1H1, PPP1R15A, GADD45A, MYC
	ncRNA metabolic process	9	6.25E-05	NOP2, NOLC1, PUS1, METTL1, TFB2M, MARS2, TFB1M, GEMIN4, DTD1
	apoptosis	9	2.56E-02	IER3, AIMP2, JUN, TNFAIP8, LGALS12, PPP1R15A, GADD45A, MYC, DDIT4
	programmed cell death	9	2.76E-02	IER3, AIMP2, JUN, TNFAIP8, LGALS12, PPP1R15A, GADD45A, MYC, DDIT4
	macromolecular complex assembly	9	4.24E-02	CYBA, HIST1H4A, NIP7, ADSL, TNPO1, PDSS1, MYC, DCTPP1, GEMIN4
Cellular component	intracellular non-membrane-bounded organelle	24	1.33E-02	GABARAPL1, NHP2L1, NIP7, PRMT3, MRPL24, NOP2, NOLC1, HIST1H4A, HMOX1, JUN, RASSF1, MRPL17, PSMC1, SNTB2, RRS1, TFB2M, CNN2, DDX21, RUVBL1, DYNC1H1, TFB1M, MYC, APEX1, GEMIN4
	non-membrane-bounded organelle	24	1.33E-02	GABARAPL1, NHP2L1, NIP7, PRMT3, MRPL24, NOP2, NOLC1, HIST1H4A, HMOX1, JUN, RASSF1, MRPL17, PSMC1, SNTB2, RRS1, TFB2M, CNN2, DDX21, RUVBL1, DYNC1H1, TFB1M, MYC, APEX1, GEMIN4
	membrane-enclosed lumen	21	2.53E-03	PHACTR3, NHP2L1, NIP7, TIMM8A, DTD1, NOP2, NOLC1, HIST1H4A, HMOX1, JUN, PSMC1, MARS2, RRS1, TFB2M, DDX21, RUVBL1, TFB1M, MYB, MYC, APEX1, GEMIN4
	intracellular organelle lumen	20	3.70E-03	PHACTR3, NHP2L1, NIP7, DTD1, NOP2, NOLC1, HIST1H4A, HMOX1, JUN, PSMC1, MARS2, RRS1, TFB2M, DDX21, RUVBL1, TFB1M, MYB, MYC, APEX1, GEMIN4
	organelle lumen	20	4.77E-03	PHACTR3, NHP2L1, NIP7, DTD1, NOP2, NOLC1, HIST1H4A, HMOX1, JUN, PSMC1, MARS2, RRS1, TFB2M, DDX21, RUVBL1, TFB1M, MYB, MYC, APEX1, GEMIN4
	nuclear lumen	16	1.39E-02	PHACTR3, NHP2L1, NIP7, NOP2, NOLC1, HIST1H4A, HMOX1, JUN, PSMC1, RRS1, DDX21, RUVBL1, MYB, APEX1, MYC, GEMIN4

(*Continued*)

Go category	Go term	Gene count	*P*-value	Gene involved
	mitochondrion	15	2.65E-03	LGALS12, SFXN4, TIMM8A, MSRB3, DTD1, MTHFD1, MRPL24, CYBA, COQ2, MRPL17, SLC25A22, MARS2, SLC25A19, TFB2M, TFB1M
	nucleolus	9	4.19E-02	NOP2, NOLC1, NHP2L1, NIP7, HMOX1, PSMC1, RRS1, DDX21, GEMIN4
Molecular function	RNA methyltransferase activity	3	7.06E-03	METTL1, TFB2M, TFB1M
	endoribonuclease activity	3	3.01E-02	RNASE2, RNASE3, APEX1
	transferase activity	3	3.25E-02	COQ2, SRM, PDSS1
	N-methyltransferase activity	3	3.75E-02	PRMT3, TFB2M, TFB1M
	rRNA methyltransferase activity	2	2.29E-02	TFB2M, TFB1M
	rRNA (adenine) methyltransferase activity	2	2.29E-02	TFB2M, TFB1M
	rRNA (adenine-N6,N6-)-dimethyltransferase activity	2	2.29E-02	TFB2M, TFB1M
	phospholipase D activity	2	3.98E-02	PLD6, HMOX1
